# The Interactive Effect of High Doses of Chromium(III) and Different Iron(III) Levels on the Carbohydrate Status, Lipid Profile, and Selected Biochemical Parameters in Female Wistar Rats

**DOI:** 10.3390/nu12103070

**Published:** 2020-10-08

**Authors:** Halina Zofia Staniek, Ewelina Król, Rafał Wojciech Wójciak

**Affiliations:** 1 Department of Human Nutrition and Dietetics, Poznan University of Life Sciences, 60-624 Poznan, Poland; ewelina.krol@up.poznan.pl; 2 Department of Clinical Psychology, Poznan University of Medical Sciences, 60-812 Poznan, Poland; rafwoj@ump.edu.pl; 3 Department of Dietetics, Faculty of Physical Culture in Gorzow Wielkopolski, Poznan University of Physical Education, 61-871 Poznan, Poland

**Keywords:** Fe deficiency, Fe excess, chromium(III), interactions, carbohydrates metabolism, biochemical parameters, rats

## Abstract

The aim of the study was to evaluate the main and interactive effects of chromium(III) propionate complex (Cr3) supplementation and different iron supply on the carbohydrate metabolism, lipid profile and other selected biochemical parameters of rats. The experiment was carried out in a two-factor design, in which rats were fed a diet with different proportions of Fe(III) and Cr(III) for six weeks. Fifty-four healthy female Wistar rats were divided into nine experimental groups with different Fe(III) levels, i.e. adequate—control group (45 mg/kg)—100% recommended daily dietary dose of Fe for rodents, deficient (5 mg/kg) and oversupply (180 mg/kg—400%). At the same time they were supplemented with Cr(III) of doses 1 (adequate), 50 and 500 mg/kg of diet. The activity and concentrations of most biochemical parameters were measured with standard enzymatic, kinetic, and colorimetric methods. HOMA-IR and QUICKI indexes were calculated according to appropriate formulas. It was found that there was an interactive effect of high Cr(III) doses and different Fe(III) levels in the diet on the carbohydrate metabolism and insulin resistance indexes. The presented results suggested that iron deficient diet fed animals led to insulin resistance; however, an effect is attenuated by Cr(III) supplementation at high doses. There were no significant changes in the rats’ lipid profile (except for the high density lipoprotein cholesterol (HDL-C) level) and most of the other biochemical parameters, such as the leptin, aspartate aminotransferase (AST), alanine transaminase (ALT), total protein (TP), creatinine (Crea) and the urea (BUN) concentrations. The study proved that the Cr(III) supplementation, independently and in combination with diversified Fe(III) content in the diet, affected the carbohydrate metabolism and insulin resistance indexes but did not affect lipid profile and most of the other biochemical parameters in healthy rats. The findings proved the role of Fe and Cr(III) and their interactions on disturbances carbohydrates metabolism.

## 1. Introduction

An inadequate supply of nutrients in the diet and their abnormal levels in the body can lead to many metabolic disorders. Minerals are significant to normal body function because they participate in numerous metabolic processes. However, the role of some elements, especially: Fe, Cr(III), Zn, Cu, and Mg in the pathogenesis of insulin resistance, diabetes, anemia, and Fe overload has not been fully elucidated and their mechanisms are not fully clear. Recent experimental and clinical trials have shown that there is a relationship between the consumption of minerals, their levels, and distribution in the body and the occurrence of metabolic disorders [1,2,3,4].

Iron deficiency is one of the most common nutritional deficiencies [5]. Statistics show that it affects about 1.5–1.8 billion people worldwide [6,7,8]. According to the WHO, 5% of the world population, 20% of menstruating women, and 30–40% of children in developed countries suffer from iron deficiency [9]. This element in the appropriate amount is necessary for the proper function of the organism. It is an ingredient of hemoglobin, myoglobin, and various enzymes involved in the transport and storage of oxygen and electron transport. Fe deficiency contributes to the reduction of oxygen transport capacity, energy production, and cell proliferation [10]. It is a frequent cause of anemia [11]. It is estimated that over 30% of the global population has anemia, mainly due to Fe deficiency [12,13]. It is mainly caused by insufficient supply of iron with diet, impaired absorption of this element, excessive blood loss in menstrual cycles, diseases such as diabetes mellitus (DM), inflammatory bowel disease (IBD), and increased physiological requirements for iron, e.g., in pregnancy and lactation [14]. 

On the other hand, researchers also pay more and more attention to the consequences of excessive iron accumulation in the body, which may be harmful [15]. The accumulation of macro- and microelements in the body is mainly caused by environmental pollution, improper diet, or metabolic disorders [16]. Iron is both naturally found and added purposely to various foods. It is also available as dietary supplements. Public health interventions, such as food fortification with iron compounds, have been undertaken to reduce the incidence of Fe deficiency anemia and to improve health. These activities as well as uncontrolled and unjustified dietary supplementation with Fe compounds arouse a lot of controversy because some groups of people are at higher risk of Fe overload due to additional exposure to Fe in the diet [15,17]. For example, predisposition to increased absorption and accumulation of iron from the diet has people with hereditary hemochromatosis [18].

Another global problem is diabetes and the costs of its treatment. It is estimated that the number of people with diabetes will have increased to 693 million by 2045 [19]. Some studies suggest a specific role of selected trace elements in the pathogenesis and progression of this disease [1,20]. Therefore, the maintenance of trace element homeostasis is essential for the regulation of numerous metabolic functions in the organism. Imbalance of trace elements may disturb proper carbohydrate and lipid metabolism or cause oxidative stress, which may contribute to insulin resistance and the development of diabetes complications [1,21,22].

It was stated that iron homeostasis is disturbed in diabetes [2,4,20,23,24,25,26,27]. There are many hypotheses concerning the mechanism of its participation in the development of diabetes. Most of the evidence supports the hypothesis that disturbed Fe homeostasis causes the overproduction of free radicals and induces oxidative stress [28,29,30].

On the other hand, trivalent chromium compounds are very popular supplements among diabetics. Since 2014, the “essentiality” of chromium has been questioned by EFSA and they no longer consider it necessary for humans and animals due to the ubiquitous nature of this element and its low dietary requirements [31,32]. Despite this, the beneficial effects of pharmacological doses of chromium(III) have been demonstrated in people with glucose metabolic disorders such as diabetes. According to McIver et al. [33], the risk of type 2 diabetes mellitus (T2DM) was lower in people consuming Cr(III) supplements. Therefore, new Cr(III) compounds, especially organic ones, are being sought. Their forms should exhibit a significant degree of absorption, safety, and efficacy, especially to improve glucose and lipid metabolism in patients with diabetes. One of these compounds is the tri-nuclear Cr(III) complex with propionic acid, aka Cr3, or CrProp. Our earlier study, as well as other authors’ studies, showed that in comparison with other Cr(III) compounds, Cr3 was characterized by a significant degree of absorption (40–60%) and it was relatively safe (LD_50_ > 2000 mg/kg body weight (b.w.)) [34,35,36,37].

It is thought that both Fe and Cr(III) may play an important role in the pathogenesis of diabetes, but their mechanisms of action are not fully understood. In vitro conditions, it was found that Fe(III) and Cr(III) are transported by the same protein—transferrin (Tf)—and can compete for binding to it. Thus, they may mutually inhibit absorption and transport to tissues [38]. These interactions may affect Fe and Cr homeostasis in the body and thus interfere with some metabolic functions. Therefore, it seems reasonable to include the interaction between these elements and their role in the etiology of the development of type 2 diabetes and cardiovascular disease. The effects of Cr(III) supplementation with an insufficient or excessive supply of Fe in vivo conditions are not known yet. In our opinion, the interaction between these elements and their effect on the development of carbohydrate and lipid metabolism disorders is also an important issue.

Therefore, the research hypothesized was that there is interaction between Fe(III) and Cr(III). The direction of this action depends on their mutual proportions in the diet and organism and determines the body’s response, which is manifested by disturbed carbohydrate and lipid metabolism.

## 2. Material and Methods

### 2.1. Test Chemicals

Chromium(III) propionate (Cr3 or CrProp) in the form of nitrate salt [Cr_3_O(O_2_CCH_2_CH_3_)_6_(H_2_O)_3_]NO_3_] was used as a source of Cr(III) in the diet. The complex was synthesized by the modified methodology described by Earnshaw et al. [39]. The Cr3 complex contained 21.5% of Cr, which was measured with a AAS-3 spectrometer with background correction (Carl-Zeiss, Jena, Germany). 

As a source of Fe(III) in the diet was used iron(III) citrate—the compound recommended for rodents (reagent grade, 16.6% Fe) [40]—which was purchased from Sigma-Aldrich (St. Louis, MO, USA).

### 2.2. Animals and Diets

The study was conducted in a two-factor design, with three different doses of Cr(III) [1 (control), 50, and 500 mg/kg] and three different levels of Fe(III) in the diet [deficient (D)—5 mg/kg, control (C)—45 mg/kg and excess (E)—180 mg/kg]. The model study was approved by the Local Animals Ethics Committee (approval number: 60/2013). The fifty-four 6-week-old female Wistar rats (*Rattus norvegicus*) were obtained from the Department of Toxicology, Poznan University of Medical Sciences, Poland.

The rats had been acclimated for 5 days before the study began. Then, the animals were divided into 9 groups, each with an initial mean body weight (b.w.) of 130.5 g. During the experiment the rats were housed in single cages, transparent under optimal and controlled living conditions (temperature 19–22 °C, air humidity 55–60%, 12 h light/12 h dark cycle). Access to diets and water were ad libitum throughout the whole study. For 6 weeks the rats were fed semi-purified AIN-93M diets composed according to the recommendations of the American Institute of Nutrition [40] and modified for the content of Fe(III) and Cr(III) in the diet. The detailed chemical composition of the experimental diets was checked and shown in Table 1.

Fifty-four healthy female Wistar rats were divided into 9 experimental groups (*n* = 6) with different Fe(III) and Cr(III) levels, as was shown in Table 2. The diet intake was recorded daily. Body weight gains were monitored weekly.

### 2.3. Data Collection

After the experiment the animals were euthanized by asphyxiation with CO_2_. Blood was drawn from the rats’ hearts and collected into tubes containing EDTA. Next, it was centrifuged (3500× *g* for 10 min, 4 °C) for further analyses. Most of the tested parameters in blood plasma were measured immediately after sampling, except for insulin, leptin, and ghrelin, which were measured later from samples frozen at −80 °C.

### 2.4. Laboratory Analyses

Chemical composition of diets was determined based on the standard assays: the protein content—the Kjeldahl method, the fat content—the Soxhlet method. The ash content was determined by burning diet samples in muffle furnace at temperature 550 °C. The diet samples for mineral analyses were digested with concentrated 65% spectra pure nitric acid (Merck) in a Microwave Digestion System MARS-5 (CEM, Matthews, NC, USA). The concentration of tested elements in mineralised samples was determined with the flame atomic absorption spectrometry (F-AAS) (AAS-3, with background correction, Carl-Zeiss, Jena, Germany).

The following standard methods were used to measure blood plasma indices with Cobas analysers (Cobas Integra 400 and 800 analysers, Roche Diagnostics (Hitachi, Tokyo, Japan). The plasma glucose concentration was measured with the hexokinase method [41]. The triglycerides (TG), total cholesterol (T-Chol), high density lipoprotein cholesterol (HDL-C) and low density lipoprotein cholesterol (LDL-C) concentrations were measured with the colorimetric methods [42,43,44]. The activity of alanine transaminase (ALT), aspartate aminotransferase (AST) and alkaline phosphatase (ALP), and the urea (BUN) concentration were measured with the kinetic methods [44,45,46]. The total protein concentration (TP) was measured with the colorimetric method, using Cu^2+^ ions [44]. The creatinine (Crea) concentration was measured with the kinetic colorimetric method [46]. The serum insulin, ghrelin, and leptin concentrations were measured with ELISA kits (EZRMI-13K and EZRL-83K, Millipore Corporation, Burlington, MA, USA; EZRGRT-91K, Linco Research, St Charles, MO, USA). The homeostatic model assessment for insulin resistance (HOMA-IR) and the quantitative insulin sensitivity check index (QUICKI) were calculated in accordance with the standard formulas from fasting plasma glucose and insulin [47,48,49].

### 2.5. Statistical Analysis

The data obtained in the study were processed with the Statistica software version 13.3 for Windows (StatSoft, Cracow, Poland) and shown as arithmetic mean ± standard deviation (SD). The results were analysed with two-way analysis of variance (two-way ANOVA/MANOA, factors of Fe(III) and Cr(III) dietary content, test F). If the two-way ANOVA indicated a significant Fe(III) content effect, Cr(III) level effect, or Fe × Cr interaction, a subsequent one-way ANOVA and post hoc comparison were applied using Tukey-test with significance set at *p* < 0.05.

## 3. Results

The effects of different levels Fe(III) and Cr(III) on the rats’ carbohydrate metabolism and insulin resistance indexes are shown in Table 3. Both the iron deficiency and excess reduced the glucose concentration (*p* < 0.05) by 16.5% and 19.1%, respectively, as compared with the control level. While the high doses of Cr(III) did not affect the glucose concentration in the experimental animals. There was no interactive effect of the experimental factors on this parameter. The diversified Fe(III) supply and Cr(III) supplementation in the diet affected both independently and in a combination the insulin concentration and insulin resistance indexes, such as HOMA-IR (Homeostatic Model Assessment of Insulin Resistance) and Quciki (Quantitative Insulin Sensitivity Check Index) (Table 3).

As the Fe(III) level in the rats’ diets increased, the insulin concentration decreased. In comparison with the control Fe level in the diet, Fe(III) deficiency significantly increased the insulin concentration (almost two times—108.5%) (*p* < 0.001), whereas Fe excess slightly reduced it. In comparison with the recommended Cr(III) level in the diet (1 mg/kg), Cr(III) supplementation at doses of 50 and 500 mg/kg increased the insulin concentration by 42.3% and 45.7%, respectively. There was also a significant interactive effect of the experimental factors on the serum insulin concentration. The highest insulin concentration was noted in the group of rats fed the Fe-deficient diet with the recommended Cr(III) level, as compared with the other groups. In comparison with the control group, this concentration was almost three times higher (2.292 ± 0.688 vs. 0.575 ± 0.095 ng/mL) (*p* < 0.001). Moreover, some tendencies were observed. In the Fe deficit groups the additional Cr(III) supplementation at doses of 50 and 500 mg/kg reduced the serum insulin levels. The Cr(III) supplementation in the groups with the recommended Fe level slightly increased the serum insulin level and in the groups with Fe excess supply did not have a definite direction of this change.

For the HOMA-IR index, it was found that both an increase Fe supply in the diet (*p* < 0.001) and Cr(III) doses (*p* < 0.001) decreased the value of this parameter. In the case of different Fe levels in the diet, a significant difference was found in the values of this index between Fe deficit and Fe excess groups (11.72 ± 8.98 vs. 4.06 ± 1.10). However, these values were not significantly different from the control group (6.79 ± 2.68). In turn, the Cr(III) doses of 50 and 500 mg/kg Cr(III) reduced the HOMA-IR index value by 42.0% and 43.3%, respectively, as compared with the group with the recommended amount of Cr(III) in the rodent diet. However, only the highest dose resulted in a statistically significant effect. The combined effect of the experimental factors on the index was also significant. The group of rats fed the Fe-deficient diet and Cr(III) at the control level had a significantly higher HOMA-IR index value than the other experimental groups. In comparison with the control group, this was an increase of 272% (21.30 ± 10.09 vs. 5.73 ± 2.17). There were some noticeable trends. In the Fe(III)-deficient and Fe(III)-excess groups Cr(III) supplementation decreased the HOMA-IR index value. Its value increased slightly in the group fed the diet with the recommended Fe level (45 mg/kg).

As the Fe(III) supply in the diet increased, so did the value of the Quicki index. The lowest value was observed in the Fe-deficient groups. It was significantly higher in the groups with the recommended amount of Fe(III) in the diet and the highest in the groups with Fe(III) excess. The dietary doses of Cr(III) 50 and 500 mg/kg increased the Quciki index value, but the effect was statistically significant only for the dose of 500 mg/kg, as compared with the recommended amount of this element in the diet. The combined effect of different Fe(III) supply with diet and simultaneous Cr(III) supplementation on Quciki index has also been found. There were significantly lower values of this index in the Fe(III)-deficient groups than in the groups with the adequate and excessive Fe(III) levels, which were supplemented with the same amounts of Cr(III). The simultaneous Cr(III) supplementation of the rats in the Fe(III)-deficient and Fe(III)-excess groups in the diet increased the values of this index. However, it tended to decrease in the groups with the recommended iron level in the diet.

Ghrelin levels were not affected by Fe(III) in diet (Table 4). However, Cr(III) supplementation at doses of 50 and 500 mg/kg reduced the level of this hormone by 20% and 19%, respectively. The serum ghrelin concentration was also affected by the interaction of Fe(III) levels and high dietary doses of Cr(III). There were significantly lower levels of this hormone in the Fe(III)-deficient and Fe(III)-excess groups supplemented with Cr(III) at a dose of 50 mg/kg and in the group with the recommended Fe level supplemented with Cr(III) at a dose of 500 mg/kg, as compared with the control group, where the level of ghrelin was the highest.

The experimental factors did not independently or in combination have any significant effect on the leptin, AST, and ALT concentrations in the rats (Table 4).

However, as the Fe(III) levels in the diet increased, the ALP levels decreased. There were significantly lower ALP levels in the rats fed the Fe-deficient diet than in the control or Fe-excess groups. The Cr(III) supplementation at a dose of 50 mg/kg reduced the value of this parameter by 12.8%, whereas at a dose of 500 mg/kg it was similar to the control group. There was no interactive effect of the experimental factors on this parameter.

As can been seen in Table 5, neither different Fe(III) levels in the diet nor high Cr(III) doses (independently or in combination) caused significant changes in the parameters of renal function, such as concentrations total protein (TP), creatinine (Crea), and urea (BUN).

In addition, neither Fe deficiency nor its excess in the diet combined with Cr(III) supplementation resulted in a significant interaction effect on the Wistar rats’ lipid profile, except the HDL-cholesterol (HDL-C) level (Table 6). Both the Fe(III) deficit and excess in the diet increased the HDL-C concentration in the female rats. Although Cr(III) supplementation on its own had no effect, there was a significant interactive effect of Cr(III) and Fe(III) on this parameter. The HDL-C level was significantly lower in the rats fed the diet with the adequate amount of Fe and supplemented with Cr(III) at a dose of 50 mg/kg, as compared with the animals fed the diet with an excessive Fe supply and the control Cr(III) level. There were no significant changes among other groups.

## 4. Discussion

Disturbed Fe homeostasis may be related to the etiology of some chronic diseases, including anemia [24], diabetes [50], cancer [51,52], and cardiovascular diseases [17]. Both the deficiency and accumulation of some microelements may stimulate a different pathway of their metabolism and affect the metabolism of other elements, which may result in the development of these diseases. Interactions between elements may also stimulate many other disorders [53]. Currently, more and more attention is paid to the potential role of chromium(III) and iron in glucose metabolism and the development of diabetes [1,3]. 

To our knowledge, this is the first time that the interactive effect of Cr(III) supplementation and different Fe(III) levels in the diet on glucose metabolism and insulin resistance indexes as well as the HDL-C concentration in Wistar rats were identified. We suppose that new and potentially relevant findings appeared from this study. Usually, these factors were analysed separately in the context of the development of diabetes. As our research shows, the interactions between these elements should be taken into account in the development of carbohydrate disorders. Our results suggest that the disturbed homeostasis of both Fe and Cr(III) (deficiency/excess) disrupts the carbohydrate metabolism that may lead to diabetes. Relations between trace element levels and the prevalence of previously undiagnosed type 2 diabetes mellitus (T2DM) were studied by Hansen et al. [1]. Their results suggested a possible role among others of chromium and iron as well as other elements (Br, Cd, Ni, Ag, and Zn) in the development of T2DM. In addition, Zhou et al. [3] analysed the Cr and Fe concentrations in the serum and urine of patients with impaired fasting glucose (IFG), impaired glucose tolerance (IGT), type 1 diabetes mellitus (T1DM), and type 2 diabetes mellitus (T2DM). They observed that the serum Cr levels were reduced in T2DM and the negative trend appeared in IFG, IGT, and T1DM. However, there were no significant differences in the serum Fe levels between these groups. They noticed a positive correlation between the serum Cr concentration and serum Fe concentration in T2DM. Meanwhile, these relations in IFG, IGT, and T1DM were not significant.

According to Ahmed et al. [2], iron deficiency significantly affected the glycaemic status of diabetic patients with iron deficiency anemia (IDA) by increasing the fasting blood glucose (FBG) and glycated hemoglobin (HbA1c) levels; however, Fe supplementation normalised these parameters. The researchers observed that IDA was highly correlated with T2DM in these patients. Similarly, Krisai et al. [20] observed that the markers of insulin resistance (insulin, FBG, HbA1c, HOMA-IR) were strongly correlated with the parameters of iron metabolism (plasma ferritin and transferrin saturation (TSAT) levels) in young and healthy subjects. However, it was suggested that Fe metabolism was mainly associated with insulin resistance and, to a lesser extent, with blood glucose concentration. These data confirm our observations on the animal model. We noticed that both Fe deficiency and oversupply led to a decrease in plasma glucose concentration in female rats. Moreover, as dietary Fe levels increased, insulin concentrations and HOMA-IR index decreased and Quicki index increased. Another study demonstrated that a low Fe supply in diet (3 mg/kg) decreased serum triglycerides and cholesterol concentrations in healthy and diabetic male rats but also improved insulin and glucose tolerance in healthy Wistar rats [54].

Iron is a transition element, which may act as an oxidant and produce reactive oxygen species (ROS). Most evidence supports the hypothesis that both Fe deficiency and overload cause the overproduction of free radicals. Thus, it causes tissue damage and increases oxidative stress [28,29,30]. According to Vieyra-Reyes et al. [30], the iron deficiency promotes oxidative stress, depending on the rat’s gender and age. However, at birth and late adolescence there was more damage to the plasma, liver, and more so the brain of male than female Wistar rats.

Furthermore, increased Fe accumulation affects insulin synthesis and secretion by pancreatic β-cells. The accumulation of Fe in the liver may cause insulin resistance by interfering with the ability of insulin to inhibit hepatic glucose production [1,29,55,56,57,58]. Another hypothesis suggests that insulin may facilitate Fe accumulation by redistributing transferrin receptors to the cell surface as well as by cellular uptake and stimulation of ferritin synthesis [20,59,60]. Although iron uptake is widely regulated, an Fe excess in the diet may increase Fe levels in tissues more than it is necessary to maintain normal erythropoiesis and metabolic function [50]. The data obtained in our study confirmed these observations, where increased tissue levels of this element were noticed [61].

Silvia et al. [62] observed that iron excess increased serum triglycerides (TG) and decreased serum glucose and HbA1c levels irrespective of the diet (standard or hyperlipidaemic). However, it did not affect the serum T-Chol concentration as well as malondialdehyde (MDA) and total antioxidants levels. These authors suggested that iron excess may have modified rats’ lipid metabolism, further changed glucose homeostasis, and increased the serum triglycerides level but not the cholesterol level. In our study, it was noticed that both iron deficiency and excess increased only HDL-C levels, but they did not cause significant changes in other elements of the lipid profile (T-Chol, LDL-C, TG).

Supplementation with organic Cr(III) compounds is part of an attractive strategy of alleviating insulin resistance. For this reason, trivalent chromium complexes may act as a relevant function in the prevention of diabetes, metabolic syndrome, and related diseases. There are many suggestions about the molecular mechanisms of chromium in alleviating insulin resistance. One of them says that Cr(III) compounds potentiate the action of insulin. Our study confirmed this fact. Another says that it enhances the insulin signalling pathway by improving the tyrosine phosphorylation of insulin and the insulin receptor tyrosine kinase activity as well AMPK activity. Another one says that Cr(III) weakens the negative regulators of the insulin signalling pathway. According to another suggestion, chromium upregulates cellular glucose uptake by increasing GLUT-4 translocation to the cell surface and reduces oxidative stress in animals with insulin resistance [1,21,63,64,65,66]. Pala et al. [67] observed that CrPic supplementation at a dose of 400 µg elemental Cr/kg of diet reduced the blood glucose, T-Chol, TG, and MDA levels and improved the GLUT-2 and GLUT-4 transporters levels in the liver and muscle of rats with chronic and acute exercise training. Another study showed that dietary chromium(III) histidinate (CrHis) supplementation decreased the glucose, T-Chol, and TG levels but increased the HDL-C level. Moreover, these effects were more efficient when CrHis was combined with biotin and exercises. However, there was no significant change in the AST and ALT levels in any of the experimental groups. The Cr(III) alone and in combination with biotin and exercises also improved the protein expression levels of IRS-1, PPAR-γ, and NF-κB in the liver and muscle of Wistar rats [68]. Jovanović et al. [69] found that the treatment with chromium-enriched yeast significantly increased IRβ, pIRS- 1Tyr632, pAkt Ser473, GLUT4, and AMPK protein expression in the insulin signaling pathway. In addition, they observed increased insulin sensitivity and better utilization of glucose in a group of Holstein calves after 70 days of Cr(III) supplementation. Chromium(phenylalanine)_3_ [Cr(pa)_3_] supplementation of obese mice at a dose 150 μg Cr/kg/b.w. for six weeks improved their glucose tolerance, as compared with untreated mice. The Cr(pa)_3_ complex enhanced insulin-stimulated phosphorylation of Akt in a time- and concentration-dependent way without changing the phosphorylation of insulin receptors [70].

Feng at al. [71] observed that long-term administration of Cr(III) malate to Sprague-Dawley rats at daily doses of 10.0, 15.0, and 20.0 µg/kg b.w. reduced the concentration of T-Chol, LDL-C, and TAG only in the male but not female animals. However, it did not affect carbohydrate metabolism parameters such as FBG, serum insulin concentrations, IR-index and related enzymes (G6PD and GCK level), and other biochemical indices (ALT, AST, ALP, TP, BUN, Crea) in rodents of both gender. Seif [72] noted that Cr supplementation of hypercholesterolemic rats at a dose of 200 µg/day CrPic for 10 weeks improved their lipid profile and reduced excessive platelet aggregation, mainly by lowering the cholesterol concentration.

In turn, the addition of chromium chloride (CrCl_3_) at doses 200 and 400 µg/kg to the diet of rabbits (pregnant rabbits, their offspring and their young rabbits) did not affect the serum T-Chol, TG, TP, and urea concentrations in all generations [73]. Zhang et al. [74] observed that the maternal low chromium level increased the serum TG, LDL-C, leptin, TNF-α, and IL-6 levels in offsprings at eight months of age. However, the normalization of the offspring diet might not be able to reverse all these effects. The authors explained this by the disturbance of the PPARγ signalling pathway in the adipose tissue, which regulates adipocyte differentiation, adipogenesis, and lipid metabolism. 

A single dose of 2000 mg/kg/b.w. of the Cr3 complex did not change the glucose concentration and lipid profile in both healthy male and female Wistar rats during a 14-day experiment [36]. In addition, high doses of the Cr3 complex (100–1000 mg/kg diet) given to female Wistar rats for six weeks were not effective on glucose levels and lipid profile (except the TAG level), hepatic enzymes activity (ALT, AST) and selected poisoning indices (TP, Crea, and BUN concentrations) [75]. Five-week supplementation of diabetic male rats with the Cr3 complex at doses of 10 and 50 mg Cr/kg diet did not significantly change their blood glucose concentration but improved their insulin sensitivity (HOMA-IR index) and decreased the serum TG, T-Chol, and LDL-C concentrations [76]. Similarly, Doddigrala et al. [77] noticed that chromium picolinate (CrPic) (1.4 µg/day) and melatonin (Mel) (200 µg/day) administered alone or in a combination significantly lowered the HOMA-IR index, T-Chol and TG levels but increased the HDL-C level. In this way, it prevented insulin resistance and T2DM in male Wistar rats fed a high carbohydrate diet (HCD). The supplementation of mice fed a high-fat diet (HFD) with a novel Grifola frondosa polysaccharide-chromium(III) complex (GFP-Cr(III)) at daily doses of 3 and 9 mg Cr(III)/kg/b.w. for eight weeks resulted in hyperglycaemic effect and prevented hyperlipidaemia and streptozotocin (STZ)-induced diabetes [78].

Other studies showed that two-week administration of different Cr(III) complexes, such as chromium methionine (CrMet), chromium rutin complex (CrRC), chromium folate complex (CrFC), and chromium stachyose complex (CrSC) to mice with alloxan-induced diabetes had beneficial effects on their glucose and lipid metabolism and resulted in hepatoprotective effect [79,80]. The observed inconsistencies may be related to the different forms, levels, and the time of supplementation with Cr(III) compounds, as well as the sex and health of the animals used in the research. Analytical problems related to biomarkers of Cr deficiency in the body also pose some difficulties in the interpretation of the obtained results.

Further research is necessary to investigate the role of selected elements and their interaction and molecular mechanisms of actions as well in the development of diabetes, metabolic syndrome, and related diseases.

## 5. Conclusions

In view of the above results, it seems that the high dietary doses of Cr(III), independently and in combination with diversified Fe(III) content in the diet, affected the carbohydrate metabolism and insulin resistance indexes, but they did not affect the lipid profile (except HDL-C) or most of the biochemical parameters of healthy rats. 

The study proved the role of Fe and Cr(III) and their mutual interactions on disturbances inter alia carbohydrates metabolism. The obtained results suggested that iron deficient fed animals led to insulin resistance; however, an effect is attenuated by Cr(III) supplementation at high doses. For this reason, further research is necessary to investigate the role of selected elements and their interaction in the development of diabetes, metabolic syndrome, and related diseases.

## Figures and Tables

**Table 1:** The chemical composition of diets (mean ± SD).

Ingredients	Unit	Content of Ingredients in Diets								
		C1	C50	C500	D1	D50	D500	E1	E50	E500
Energy	MJ 100/g	1.82 ± 0.00	1.83 ± 0.03	1.89 ± 0.06	1.92 ± 0.05	1.80 ± 0.04	1.87 ± 0.03	1.88 ± 0.04	1.82 ± 0.03	1.81 ± 0.02
Fat	%	7.46 ± 0.05	7.22 ± 0.08	7.26 ± 0.31	7.36 ± 0.24	6.62 ± 0.29	7.19 ± 0.10	7.31 ± 0.10	7.11 ± 0.06	6.83 ± 0.05
Protein	%	17.12 ± 0.10	17.17 ± 0.14	17.27 ± 0.24	17.64 ± 0.35	17.90 ± 0.13	17.22 ± 0.16	17.42 ± 0.30	17.01 ± 0.12	17.72 ± 0.39
Carbohydrates	%	63.46	63.67	63.45	63.55	64.12	63.78	64.05	63.11	64.77
Dry Mass	%	90.47 ± 0.05	90.54 ± 0.22	90.26 ± 0.08	90.14 ± 0.26	89.40 ± 0.03	89.70 ± 0.14	89.99 ± 0.36	89.67 ± 0.54	89.90 ± 0.23
Ash	%	2.47 ± 0.04	2.48 ± 0.11	2.32 ± 0.50	2.45 ± 0.08	2.77 ± 0.05	2.63 ± 0.12	2.36 ± 0.21	2.67 ± 0.08	2.58 ± 0.08
Ca	g/kg	4.96 ± 0.13	5.16 ± 0.13	5.02 ± 0.11	5.00 ± 0.19	5.10 ± 0.11	5.02 ± 0.37	5.17 ± 0.02	5.09 ± 0.11	4.94 ± 0.17
Mg	mg/kg	441.42 ± 8.99	473.12 ± 1.44	511.73 ± 17.55	478.69 ± 15.46	473.73 ± 56.11	529.95 ± 13.13	503.86 ± 23.80	501.62 ± 19.90	529.98 ± 15.50
Fe	mg/kg	58.05 ± 0.70	57.09 ± 2.83	59.13 ± 1.98	3.43 ± 0.38	3.30 ± 1.08	3.03 ± 0.59	218.12 ± 7.53	207.55 ± 21.49	229.78 ± 13.04
Cr	mg/kg	1.24 ± 0.23	50.04 ± 6.48	425.14 ± 10.28	1.69 ± 0.12	48.89 ± 2.00	459.14 ± 24.42	1.95 ± 0.61	49.42 ± 5.40	431.59 ± 14.82
Zn	mg/kg	52.51 ± 1.60	50.71 ± 1.90	52.41 ± 2.02	49.26 ± 9.70	45.90 ± 7.51	44.41 ± 5.66	44.80 ± 4.21	40.86 ± 0.49	43.13 ± 3.00
Cu	mg/kg	5.45 ± 0.94	4.43 ± 0.15	5.93 ± 0.79	5.11 ± 0.88	5.53 ± 0.90	5.51 ± 0.60	5.82 ± 0.71	6.28 ± 0.20	6.62 ± 1.02

**Table 2:** Model of the experiment.

Factor	Groups	*N* (54)	Level of Factor
Factor **A** Fe Level in Diet	**D**	**18**	A1—10% recommended Fe level in the diet for rodents (5 mg/kg) (Fe—deficiency)
	**C**	**18**	A2—recommended Fe level in the diet for rodents (45 mg/kg) (Fe—control)
	**H**	**18**	A3—400% recommended Fe level in the diet for rodents (180 mg/kg) (Fe—oversupply)
Factor **B** Cr(III) Level in Diet	**1**	**18**	B1—recommended Cr(III) level in the diet for rodents (1 mg/kg) (Cr—control dose)
	**50**	**18**	B2—I supplemental dose of Cr(III) (50 mg/kg) (Cr—medium dose)
	**500**	**18**	B3—II supplemental dose of Cr(III) (500 mg/kg) (Cr—high dose)
**Combinations Factors**		***N*** **(54)**	
**A1B1**	**D1**	**6**	Fe 5 mg/kg, Cr 1 mg/kg
**A1B2**	**D50**	**6**	Fe 5 mg/kg, Cr 50 mg/kg
**A1B3**	**D 500**	**6**	Fe 5 mg/kg, Cr 500 mg/kg
**A2B1**	**C1 (control)**	**6**	Fe 45 mg/kg, Cr 1 mg/kg
**A2B2**	**C50**	**6**	Fe 45 mg/kg, Cr 50 mg/kg
**A2B3**	**C500**	**6**	Fe 45 mg/kg, Cr 500 mg/kg
**A3B1**	**H1**	**6**	Fe 180 mg/kg, Cr 1 mg/kg
**A3B2**	**H50**	**6**	Fe 180 mg/kg, Cr 50 mg/kg
**A3B3**	**H500**	**6**	Fe 180 mg/kg, Cr 500 mg/kg

**Table 3:** The effects of the Fe(III) and Cr(III) levels in the diet on the glucose and insulin concentrations and parameters of insulin resistance in rats.

Factors	Factor A Level (mg/kg)	Factor B Level (mg/kg)		*N*	Parameters							
			Group		Glucose Concentration (mg/dL) (mMol/L)	*p*	Insulin (ng/mL) (mU/L)	*p*	HOMA-IR Index	*p*	Quicki (Quantitative Insulin Sensitivity Check Index)	*p*
					Main Effects (Mean ± SD)							
Fe-deficiency	5		D	18	147.78 ± 29.75 ^a^ (8.203 ± 1.652)	*p* < 0.05	1.274 ± 0.837 ^b^ (31.625 ± 20.777)	*p* < 0.001	11.72 ± 8.97 ^b^	*p* < 0.001	0.280 ± 0.020 ^a^	*p* < 0.001
Fe-control	45		C	18	176.89 ± 44.80 ^b^ (9.819 ± 2.487)		0.611 ± 0.109 ^a^ (15.167 ± 2.715)		6.79 ± 2.68 ^a,b^		0.294 ± 0.015 ^b^	
Fe-oversupply	180		H	18	143.11 ± 37.93 ^a^ (7.944 ± 2.105)		0.464 ± 0.048 ^a^ (11.531 ± 1.203)		4.06 ± 1.10 ^a^		0.312 ± 0.010 ^c^	
Cr-control dose		1	1	18	156.28 ± 45.21 (8.675 ± 2.510)	NS	1.108 ± 0.942 ^b^ (27.512 ± 23.379)	*p* < 0.001	10.53 ± 9.69 ^b^	*p* < 0.001	0.289 ± 0.027 ^a^	*p* < 0.05
Cr-medium dose		50	50	18	154.11 ± 39.49 (8.554 ± 2.192)		0.639 ± 0.175 ^a^ (15.853 ± 4.357)		6.07 ± 2.28 ^a,b^		0.298 ± 0.015 ^a,b^	
Cr-high dose		500	500	18	157.39 ± 37.83 (8.736 ± 2.100)		0.602 ± 0.150 ^a^ (14.957 ± 3.716)		5.97 ± 2.73 ^a^		0.299 ± 0.015 ^b^	
**Factor Combinations**					**Interaction Effects** **(Mean ± SD)**							
Fe-deficiency	5	1	D1	6	148.00 ± 37.27 (8.215 ± 2.069)	NS	2.292 ± 0.688 ^b^ (56.903 ± 17.074)	*p* < 0.001	21.30 ± 10.09 ^b^	*p* < 0.001	0.257 ± 0.013 ^a^	*p* < 0.001
	5	50	D50	6	156.00 ± 32.65 (8.659 ± 1.812)		0.815 ± 0.159 ^a^ (20.239 ± 3.935)		7.70 ± 1.68 ^a^		0.287 ± 0.009 ^b^	
	5	500	D500	6	139.33 ± 19.69 (7.734 ± 1.093)		0.714 ± 0.104 ^a^ (17.731 ± 2.593)		6.17 ± 1.60 ^a^		0.293 ± 0.013 ^b,c^	
Fe-control	45	1	C1	6	158.83 ± 43.65 (8.816 ± 2.423)		0.575 ± 0.095 ^a^ (14.279 ± 2.370)		5.73 ± 2.17 ^a^		0.300 ± 0.016 ^b,c^	
	45	50	C50	6	179.50 ± 48.09 (9.964 ± 2.669)		0.614 ± 0.100 ^a^ (15.235 ± 2.480)		6.77 ± 2.09 ^a^		0.293±0.013 ^b,c^	
	45	500	C500	6	192.33 ± 43.83 (10.676 ± 2.433)		0.644 ± 0.137 ^a^ (15.988 ± 3.395)		7.86 ± 3.56 ^a^		0.289±0.014 ^b^	
Fe-oversupply	180	1	H1	6	162.00 ± 59.39 (8.992 ± 3.296)		0.457 ± 0.044 ^a^ (11.355 ± 1.095)		4.56 ± 1.74 ^a^		0.309±0.016 ^b,c^	
	180	50	H50	6	126.83 ± 15.90 (7.040 ± 0.883)		0.487 ± 0.061 ^a^ (12.086 ± 1.522)		3.75 ± 0.39 ^a^		0.315±0.005 ^c^	
	180	500	H500	6	140.50 ± 18.92 (7.799 ± 1.050)		0.449 ± 0.037 ^a^ (11.152 ± 0.910)		3.88 ± 0.70 ^a^		0.314±0.008 ^c^	

Note: The values which do not have the same superscript letter are significantly different (*p* < 0.05); NS: no significant effect; SD: standard deviation.

**Table 4:** The effects of the Fe(III) and Cr(III) levels in the diet on the concentrations of selected hormones and enzymes in rats.

Factors	Factor A Level (mg/kg)	Factor B Level (mg/kg)		*N*	Parameters									
			Group		Ghrelin (ng/mL)	*p*	Leptin (ng/mL)	*p*	AST (U/L)	*p*	ALT (U/L)	*p*	ALP (U/L)	*p*
					Main Effects (Mean ± SD)									
Fe-deficiency	5		D	18	0.361 ± 0.098	NS	4.406 ± 0.816	NS	133.063 ± 45.543	NS	21.125 ± 8.724	NS	97.294 ± 13.284 ^b^	
Fe-control	45		C	18	0.459 ± 0.179		4.910 ± 1.077		173.882 ± 64.405		22.000 ± 6.055		75.611 ± 11.003 ^a^	*p* < 0.001
Fe-oversupply	180		H	18	0.400 ± 0.135		4.327 ± 1.010		138.333 ± 68.526		19.067 ± 9.838		70.667 ± 12.054 ^a^	
Cr-control dose		1	1	18	0.468 ± 0.146 ^b^	*p* < 0.05	4.649 ± 1.204	NS	163.278 ± 63.889	NS	24.056 ± 10.619	NS	85.667 ± 19.629 ^b^	
Cr-medium dose		50	50	18	0.374 ± 0.155 ^a^		4.839 ± 0.923		133.000 ± 47.129		19.924 ± 5.543		74.667 ± 13.750 ^a^	*p* < 0.05
Cr-high dose		500	500	18	0.378 ± 0.116 ^a^		4.168 ± 0.741		150.769 ± 74.458		17.917 ± 5.869		82.412 ± 14.573 ^a,b^	
**Factor Combinations**					**Interaction Effects** **(Mean ± SD)**									
Fe-deficiency	5	1	D1	6	0.443 ± 0.066 ^a,b,c^	*p* < 0.01	4.185 ± 0.628	NS	161.833 ± 57.957	NS	26.667 ± 12.061	NS	107.000 ± 14.233	
	5	50	D50	6	0.311±0.091 ^a,b^		4.984 ± 0.913		119.833 ± 29.607		18.333 ± 3.445		87.500 ± 10.426	
	5	500	D500	6	0.328±0.086 ^a,b,c^		4.011 ± 0.590		109.750 ± 24.116		17.000 ± 4.320		96.400 ± 2.302	
Fe-control	45	1	C1	6	0.556±0.190 ^c^		5.414 ± 1.460		156.333 ± 58.157		20.833 ± 7.600		75.333 ± 13.227	
	45	50	C50	6	0.502 ± 0.180 ^a,b,c^		4.575 ± 0.841		164.000 ± 47.397		24.000 ± 4.183		72.500 ± 11.149	NS
	45	500	C500	6	0.320 ± 0.058 ^a,b^		4.741 ± 0.793		199.667 ± 82.638		21.400 ± 6.348		79.000 ± 9.252	
Fe-oversupply	180	1	H1	6	0.405 ± 0.130 ^a,b,c^		4.270 ± 1.049		171.667 ± 83.617		24.667 ± 12.612		73.838 ± 6.735	
	180	50	H50	6	0.310 ± 0.109 ^a,b^		4.958 ± 1.110		120.333 ± 55.428		16.333 ± 6.218		64.000 ± 8.837	
	180	500	H500	6	0.485 ± 0.120 ^a,b,c^		3.753 ± 0.511		107.667 ± 44.658		13.333 ± 4.163		74.167 ± 17.279	

Note: The values which do not have the same superscript letter are significantly different (*p* < 0.05); NS: no significant effect; SD: standard deviation.

**Table 5:** The effects of the Fe(III) and Cr(III) levels in the diet on the concentrations of selected biochemical parameters in rats.

Factors	Factor A Level (mg/kg)	Factor B Level (mg/kg)		*N*	Parameters					
			Group		Total Protein (TP) Concentration (g/dL)	*p*	Creatinine (Crea) Concentration (mg/dL)	*p*	Urea (BUN) Concentration (mg/dL)	*p*
					Main Effects (Mean ± SD)					
Fe-deficiency	5		D	18	6.383 ± 0.215	NS	0.509 ± 0.053 ^a,b^	*p* < 0.05	44.000 ± 9.266	NS
Fe-control	45		C	18	6.456 ± 0.273		0.537 ± 0.071 ^b^		40.750 ± 8.745	
Fe-oversupply	180		H	18	6.382 ± 0.227		0.486 ± 0.037 ^a^		38.333 ± 7.104	
Cr-control dose		1	1	18	6.361 ± 0.212	NS	0.523 ± 0.061	NS	43.588 ± 10.087	NS
Cr-medium dose		50	50	18	6.394 ± 0.277		0.509 ± 0.065		38.625 ± 7.429	
Cr-high dose		500	500	18	6.467 ± 0.222		0.501 ± 0.050		40.412 ± 7.500	
**Factor Combinations**					**Interaction Effects** **(Mean ± SD)**					
Fe-deficiency	5	1	D1	6	6.383 ± 0.183	NS	0.532 ± 0.061	NS	40.600 ± 12.818	NS
	5	50	D50	6	6.333 ± 0.250		0.513 ± 0.062		44.400 ± 7.829	
	5	500	D500	6	6.433 ± 0.234		0.482 ± 0.021		46.500 ± 7.609	
Fe-control	45	1	C1	6	6.317 ± 0.232		0.528 ± 0.078		45.500 ± 10.895	
	45	50	C50	6	6.500 ± 0.358		0.545 ± 0.075		37.600 ± 7.127	
	45	500	C500	6	6.550 ± 0.187		0.537 ± 0.071		38.200 ± 5.762	
Fe-oversupply	180	1	H1	6	6.383 ± 0.248		0.510 ± 0.049		44.167 ± 7.859	
	180	50	H50	6	6.340 ± 0.207		0.462 ± 0.013		34.667 ± 4.761	
	180	500	H500	6	6.417 ± 0.256		0.483 ± 0.023		36.167 ± 4.956	

Note: The values which do not have the same superscript letter are significantly different (*p* < 0.05); NS: no significant effect; SD: standard deviation.

**Table 6:** The effects of the Fe(III) and Cr(III) levels in the diet on the lipid profile in rats.

Factors	Factor A Level (mg/kg)	Factor B Level (mg/kg)		*N*	Parameters							
			Group		Triglycerides (TG) Concentration (mg/dL)	*p*	Total Cholesterol Concentration (mg/dL)	*p*	LDL Cholesterol (mg/dL)	*p*	HDL Cholesterol (mg/dL)	*p*
					Main Effects (Mean ± SD)							
Fe-deficiency	5		D	18	38.375 ± 11.372	NS	84.278 ± 11.876	NS	44.193 ± 7.771	NS	29.205 ± 1.893 ^b^	*p* < 0.05
Fe-control	45		C	18	46.118 ± 10.879		81.333 ± 12.049		44.858 ± 10.884		27.634 ± 2.272 ^a^	
Fe-oversupply	180		H	18	43.000 ± 9.507		90.833 ± 20.709		48.318 ± 11.981		29.219 ± 2.683 ^b^	
Cr-control dose		1	1	18	44.647 ± 11.113	NS	87.889 ± 27.357	NS	44.737 ± 12.250	NS	29.428 ± 2.830	NS
Cr-medium dose		50	50	18	41.412 ± 11.125		80.278 ± 14.776		43.155 ± 9.330		27.878 ± 2.126	
Cr-high dose		500	500	18	41.625 ± 10.682		88.278 ± 14.228		48.491 ± 8.589		28.515 ± 1.950	
**Factors Combinations**					**Interaction Effects** **(Mean ± SD)**							
Fe-deficiency	5	1	D1	6	38.000 ± 7.714	NS	90.000 ± 10.526	NS	43.613 ± 2.867	NS	30.586 ± 1.151 ^a,b^	*p* < 0.05
	5	50	D50	6	36.500 ± 8.939		77.000 ± 13.221		42.223 ± 10.164		28.370 ± 2.210 ^a,b^	
	5	500	D500	6	41.000 ± 17.649		85.833 ± 9.390		46.550 ± 7.831		28.750 ± 1.712 ^a,b^	
Fe-control	45	1	C1	6	47.167 ± 13.029		80.000 ± 14.873		43.348 ± 13.019		27.218 ± 3.007 ^a,b^	
	45	50	C50	6	48.800 ± 13.971		73.167 ± 3.251		36.756 ± 3.855		26.488 ± 1.410 ^a^	
	45	500	C500	6	42.833 ± 5.601		90.833 ± 8.448		53.120 ± 6.883		29.197 ± 1.345 ^a,b^	
Fe-oversupply	180	1	H1	6	47.667 ± 10.727		93.667 ± 23.922		47.945 ± 18.446		30.920 ± 2.368 ^b^	
	180	50	H50	6	40.167 ± 8.635		90.667 ± 18.811		52.553 ± 5.148		29.348 ± 2.031 ^a,b^	
	180	500	H500	6	40.800 ± 8.585		88.167 ± 22.666		44.458 ± 10.752		27.416 ± 2.644 ^a,b^	

Note: The values which do not have the same superscript letter are significantly different (*p* < 0.05); NS: no significant effect; SD: standard deviation.
